# The roots of *Atractylodes japonica* Koidzumi promote adipogenic differentiation via activation of the insulin signaling pathway in 3T3-L1 cells

**DOI:** 10.1186/1472-6882-12-154

**Published:** 2012-09-14

**Authors:** Yunkyung Han, Hyo Won Jung, Yong-Ki Park

**Affiliations:** 1 Oriental Medicine R&D Center, Dongguk University, Gyeongju, 780-714, Republic of Korea; 2 Department of Herbology, College of Oriental Medicine, Dongguk University, Gyeongju, 780-714, Republic of Korea

**Keywords:** *Atractylodes japonica*, Atractylodis Rhizoma Alba, 3T3-L1 adipocyte, PPARγ, GLUT4, Insulin, Type 2 diabetes

## Abstract

**Background:**

Type 2 diabetes (T2D) is a complex metabolic disorder characterized by insulin resistance and hyperglycemia. Peroxisome proliferator-activated receptor gamma (PPARγ) is a key transcription factor and plays an important role in the regulation of genes involved in adipogenic differentiation, glucose metabolism and insulin signal transduction.

**Methods:**

In this study, the effects of the root extract of *Atractylodes japonica* Koidzumi **(**Atractylodis Rhizoma Alba, ARA) on the differentiation of 3T3-L1 preadipocytes and the possible mechanism of glucose transport were investigated. 3T3-L1 cells were cultured with insulin and ARA extract.

**Results:**

In 3T3-L1 cells, ARA extract significantly enhanced adipogenic differentiation and upregulated the expression of PPARγ genes and protein in a dose-dependent manner. ARA also promoted glucose transport by increasing the glucose transporter 4 (GLUT-4), phosphatidylinositol 3-kinase (PI3K) and insulin receptor substrates-1 (IRS-1) levels.

**Conclusion:**

Our results suggest that ARA extract may be an attractive therapeutic agent for managing T2D via promoting the differentiation of adipocytes with the upregulation of PPARγ levels and the activation of the insulin signaling pathway.

## Background

The modern lifestyle, with its abundant nutrient supply and sedentary behavior, has resulted in dramatic increases in the rates of obesity-associated diseases including type 2 diabetes (T2D) [1]. T2D is a complex metabolic disorder characterized by insulin resistance and hyperglycemia [2]. Insulin resistance is a major risk factor of T2D and refers to a state in which physiological concentrations of insulin are poorly effective [3]. Insulin levels increase to compensate for progressing insulin resistance leading to decreased glucose uptake and glucose utilization [4]. Therefore, insulin resistance in target tissues, including the adipose tissue, muscle and liver, is the central pathophysiological event in the development of T2D [5].

Adipocytes are key inducers of insulin resistance that modulate adipokine secretion in T2D [6]. Abnormal differentiation of preadipocytes affects glucolipid metabolism and induces insulin resistance [7]. Adipocyte differentiation is a complex process that involves expression of several adipocyte-specific genes, including PPARγ (peroxisome proliferator-activated receptor-gamma), which lead to morphological changes and lipid accumulation within the cells [8]. PPARγ, which is mainly found in adipose tissue, is a key transcription factor that regulates adipogenesis and plays an important role in the regulation of genes involved in adipocyte differentiation, lipid storage, glucose metabolism and insulin signal transduction [6,9]. PPARγ activation can increase the number of small adipocytes, which are more sensitive to insulin, and reduce the number of large adipocytes [10]. In this regard, PPARγ has been an attractive target for new drug discovery, and several types of PPARγ agonists with new structures have been developed.

Insulin signaling involves a cascade of events initiated by insulin binding to its cell surface receptor, followed by receptor autophosphorylation and activation of receptor tyrosine kinases, resulting in tyrosine phosphorylation of insulin receptor substrates (IRS) [11]. IRS-1 is integrated with PI3-kinase (phosphatidylinositol 3-kinase), which is necessary but not sufficient for the stimulation of the glucose transporter (GLUT)-4-mediated increase in glucose transport [12]. The final step in this signaling cascade is the translocation of GLUT4 from intracellular compartments to the plasma membrane, thereby facilitating the entry of glucose into insulin-sensitive cells like fat cells [4].

The root of *Atractylodes japonica* Koidzumi (Atractylodis Rhizoma Alba, ARA) is an herbal medicine traditionally used in East Asia for treatment of obesity and related complications [13]. In recent years, several studies have reported that ARA has various pharmacological activities such as anti-obesity [8], anti-inflammatory [14], gastroprotective [15] and anti-oxidant [16] effects. However, the mechanism of its anti-diabetic effects has not yet been investigated.

Thus, in this study, to evaluate the potential anti-diabetic effects of ARA, 3T3-L1 preadipocytes were differentiated into adipocytes. The specific aim of this study was to verify the action mechanism underlying the beneficial effect of ARA on lipid accumulation, adipocytes differentiation and insulin signaling pathway, with focus on the extent of treatment during adipocytes differentiation.

## Methods

### Preparation of ARA extract

The roots of *A. japonica* Koidzumi (ARA) were purchased from the Kwanmyungdang Medicinal Herbs (Ulsan, South Korea) and authenticated by Prof. Yong-Ki Park, a medicinal botanist, as an author in this study. Voucher specimens (ARA-W-1201) have been deposited in the herbarium of Oriental Medicine R&D Center, Dongguk University, South Korea. The dried roots (210 g) were extracted by boiling in water for 3 h, filtered through a two-layer mesh and concentrated in a boiling water bath to obtain the residue (yields of 26%). ARA extract was stored at −20°C until the experiment was performed.

### 3T3-L1 cell culture and differentiation into adipocytes

3T3-L1 cells, murine preadipocytes (ATCC, Manassas, VA, USA) were maintained in Dulbecco’s Modified Eagle’s Medium (DMEM) supplemented with 10% heat-inactivated bovine calf serum (BCS; Hyclone, Logan, UT) in a 5% CO_2_ humidified atmosphere at 37°C. To differentiate preadipocytes into adipocytes, the cells were seeded in 60-mm dishes at a density of 1 × 10^6^/mL cells, and then cultured to confluence for 8 days while changing the medium every 2 days, followed by culturing for 2 days in medium supplemented with 0.5 mM 3-isobutyl-1-methylxanthine (IBMX), 1 μM dexamethasone and 5 μg/mL insulin. The cells were cultured for an additional 1 day in medium containing 5 μg/mL insulin. ARA extract was dissolved in adipocyte-induction media and filtered through 0.2 μm-pore syringe filters. The cells were treated every 2 days with ARA extract at concentrations of 100, 250 and 500 μg/mL in adipocyte-induction media for 6 days.

### MTT assay

Cell viability was assessed by the conversion of MTT [3-(4,5-dimethylthiazol-2-yl)-2,5-diphenyltetrazolium bromide] (MTT; Roche, Mannheim, Germany) to formazan. 3T3-L1 cells were pre-treated with ARA extract at different concentrations for 8 days. At the termination of culture, 10 μL of MTT solution was added to each well, and the cells were then cultured for 4 h. 100 μL of DMSO was added to each well, and then the optical density (OD) was measured at 550 nm by a microplate reader (GENios, TEKAN Instruments, Inc., Austria).

### Oil Red O staining

3T3-L1 cells were washed with 1× phosphate-buffered saline (PBS) and fixed with 10% formalin-PBS solution for 1 h. After removing this solution, the differentiated cells were stained with Oil Red O dye (Sigma Aldrich, St. Louis, MO) for 30 min at room temperature. The cells were washed four times with distilled water. Images were collected using an Olympus microscope (Tokyo, Japan). Stained oil droplets were dissolved in isopropyl alcohol and quantified at 520 nm using a microplate reader (GENios, TEKAN Instruments, Inc., Austria).

### Reverse transcription polymerase chain reaction (RT-PCR)

Total RNA from the cells was isolated with TRIzol reagent (Invitrogen, Carlsbad, CA, USA). Total RNA was reverse transcribed for 1 h at 42°C in a reaction mixture containing RNA, 1× reverse transcriptase buffer (Promega, Madison, WI), 0.5 mM of dNTP (deoxynucleotide triphosphate), 3 mM MgCl_2_, 5 U RNase inhibitor (Amersham, Piscataway, NJ), 0.5 μM oligo-dT primer, and 5 U of Superscript Reverse Transcriptase (Promega, Madison, WI) in a total volume of 20 μL. The PCR was performed using the prepared cDNA as a template with the following cycle parameters: 94°C, 2 min, 30–35 cycles; 94°C, 30s; 56 ~ 59°C, 30s; 72°C, 1 min; 92°C, 10 min. PCR products were then resolved on 1% agarose gels at 100 V. Specific genes were verified by assessing their predicted sizes under UV light. The primer sequences for PPARγ and GAPDH were as follows: PPARγ [accession no. NM 011146] Fw: 5′-GAA AGA CAA CGG ACA AAT CAC C-3′ and Rv: 5′-GGG GGT GAT ATG TTT GAA CTT G-3′, and GAPDH [accession no. XM 994067.2] Fw: 5′-CTC CTG GAG TCT ACT GGT GT-3′ and Rv: 5′-GTC ATC ATA CTT GGC AGG TT-3′. GAPDH was used as an internal control for PCR.

### Western blot

The cells were lysed with lysis buffer containing10 mM Tris–HCl, pH 7.9, 10 mM NaCl, 3 mM MgCl_2_, and 1% NP-40. After centrifugation at 12,000 rpm for 10 min, the supernatant was stored at −80°C until use. The protein concentration was determined by Bradford’s assay. 30 μg/mL of protein were separated by 8% SDS-PAGE and then transferred to nitrocellulose membranes. The membranes were blocked with 5% skim milk (BD, Franklin Lakes, NJ, USA) in TBS-T buffer (10 mM Tris–HCl, 150 mM NaCl, and 0.5% Tween-20) for 1 h. The membranes were incubated overnight with primary antibodies at 4°C and then incubated with horseradish peroxidase (HRP)-conjugated secondary antibodies. The blots were developed with ECL Western detection reagents (Amersham Bioscience, Piscataway, NJ). The antibodies used in this study were anti-PPARγ (1:1000, Santa Cruz Biotechnology, Santa Cruz, CA), anti-GLUT4 (1:500, Santa Cruz Biotechnology), anti-PI3K (1:1000, Cell Signaling Technology, Beverly, MA), anti-IRS-1 (1:500, Cell Signaling Technology), anti-phospho-IRS-1 (1:500, Cell Signaling Technology), anti-β-actin (1:1000, Sigma Aldrich) and HRP-labeled anti-rabbit or mouse IgG (1:5000; Santa Cruz Biotechnology).

### Immunofluorescence staining

The cells were grown on glass coverslips in 2-well culture plates. After 8-day treatment with ARA extract, the cells were fixed with 4% paraformaldehyde for 30 min, washed with PBS, and then permeabilized with 0.2% Triton X-100 in 1× PBS for 15 min at room temperature. After blocking in 1% BSA in 1× PBS for 1 h, the cells were incubated overnight with anti-GLUT-4 (1:50, Cell Santa Cruz Biotechnology) antibody at 4°C. Coverslips were washed and incubated with Alexa Fluor-conjugated goat anti-rabbit antibody (1:50, Santa Cruz Biotechnology) for 2 h at RT. After DAPI staining, the coverslips were mounted on glass slides and examined under a fluorescence microscope (Olympus, Japan).

### Statistical analysis

Data of all experiments are expressed as the mean ± S.D. and are representative of three independent experiments. Statistical analysis was carried out by one-way ANOVA with the Post-Hoc test using Graphpad Prism 5.0 statistical analysis software (GraphPad Software, Inc., San Diego, CA). Values of p < 0.05 were considered significant.

## Results

### Effect of ARA extract on cell viability

**Figure 1:**
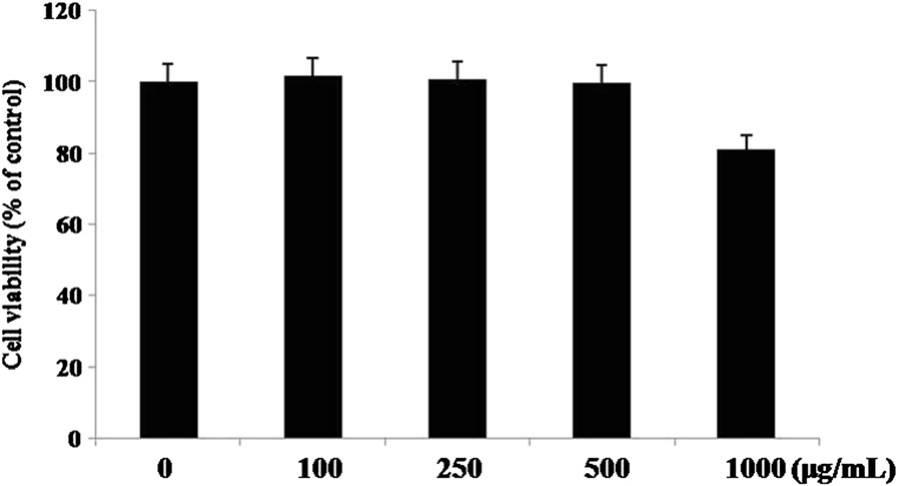
**Effect of ARA extract on cell viability in 3T3-L1 cells.** The cells were treated with or without ARA extract at concentrations of 100, 250, 500, and 1000 μg/mL for 8 days. Cell viability was determined by MTT assay. Each value represents the mean ± S.D. (*n* = 3).

To avoid any cytotoxicity caused by ARA extract, we first investigated the effect of ARA extract on the cell viability in 3T3-L1 cells by MTT assay (Figure 1). ARA extract at concentrations of 100, 250 and 500 μM did not cause cell toxicity. Therefore, we used ARA extract at concentrations of 500 μM or less for subsequent studies of its anti-diabetic properties and action mechanism in the cells.

### Effect of ARA extract on adipogenic differentiation

**Figure 2:**
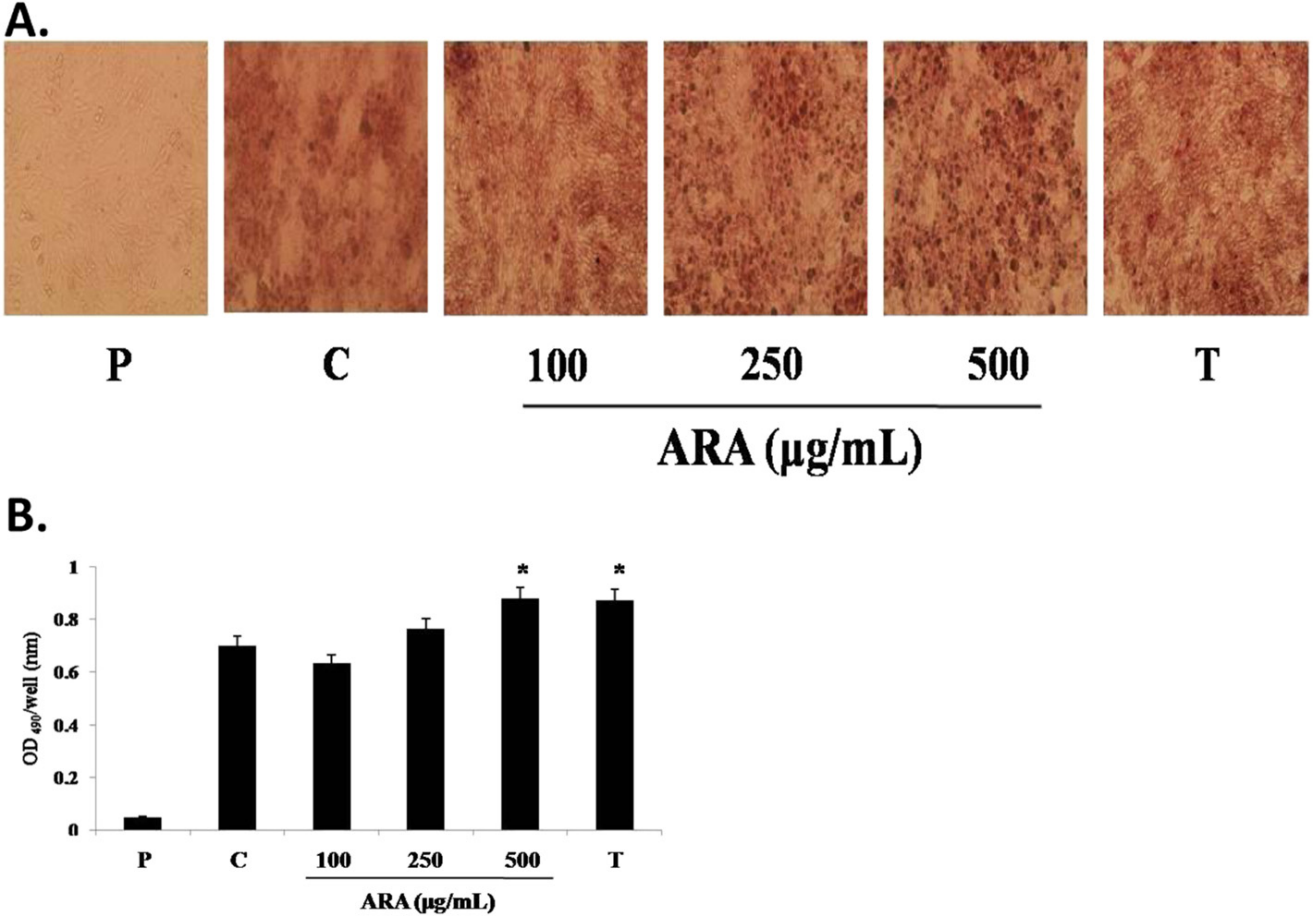
**Effect of ARA extract on lipid accumulation in 3T3-L1 cells.** The cells were induced to differentiate into adipocytes with MDI medium with or without ARA extract (100, 200 and 500 mg/mL) and troglitazone (10 μg/mL) for 8 days. Troglitazone used as a positive control. (**A**) The cells were stained with Oil Red O on day 8. Representative photomicrographics (X 100) are shown for each treatment group. (**B**) To quantify lipid accumulation in the cells, Oil Red O dye was dissolved in isopropanol and the optical density was measured at 490 nm. Data was based on the OD values. Each value represents the mean ± S.D. (*n* = 3). *p < 0.05 vs. control. P: preadipocyte, C: control, 100: ARA 100 μg/mL, 250: ARA 250 μg/mL, 500: ARA 500 μg/mL and T: troglitazone 10 μg/mL.

To investigate the effect of ARA extract on adipogenic differentiation in preadipocytes, post-confluent 3T3-L1 cells were maintained in MDI media and then treated with ARA extract at concentrations of 100, 250, and 500 μg/mL. Adipogenic differentiation was measured by Oil Red O staining for lipid droplet accumulation. As shown in Figure 2, ARA extract significantly enhanced adipogenic differentiation in 3T3-L1 cells. This effect was similar to that seen in response to troglitazone, a PPARγ agonist, in 3T3-L1 cells. This data suggests that ARA extract promotes adipogenic differentiation in preadipocytes.

### Effect of ARA extract on PPAR γ expression

**Figure 3:**
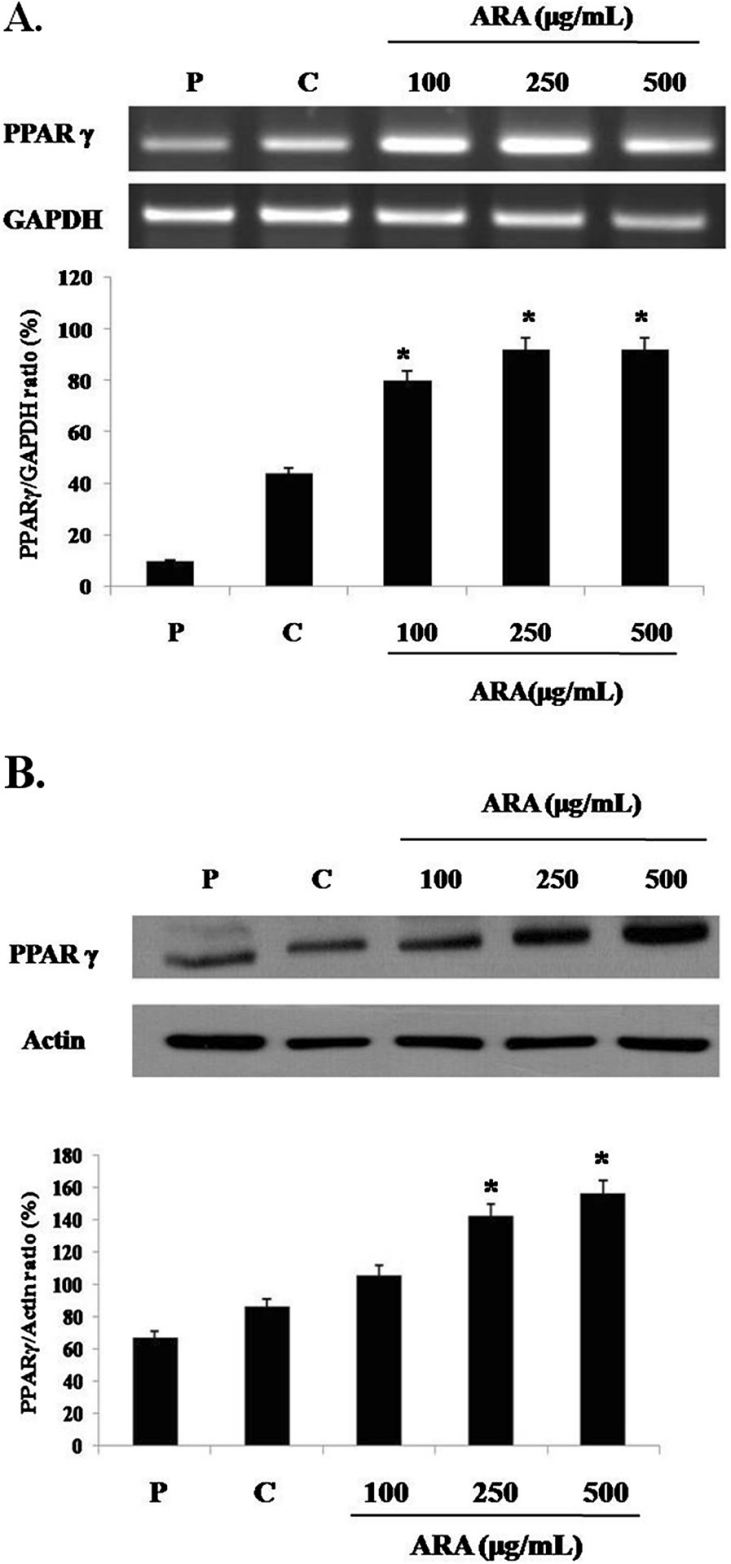
**Effect of ARA extract on PPARγ expression in 3T3-L1 cells.** The cells were induced to differentiate into adipocytes with MDI medium with or without ARA (100, 200 and 500 mg/mL) extract for 8 days. (**A**) The expression of PPARγ mRNA was analyzed by RT-PCR. Relative density was calculated as the ratio of PPARγ expression to GAPDH expression. (**B**) The protein expression of PPARγ was analyzed by western blot. Relative density was calculated as the ratio of PPARγ expression to actin. Each value represents the mean ± S.D. (*n* = 3). *p < 0.05 vs. control. P: preadipocyte, C: control, 100: ARA 100 μg/mL, 250: ARA 250 μg/mL and 500: ARA 500 μg/mL.

Adipogenic differentiation-induced lipid accumulation is accompanied by induction of the master adipogenic transcription factor, PPARγ, in adipocytes [6]. Therefore, we investigated the expression of PPARγ mRNA and protein in 3T3-L1 adipocytes. As shown in Figure 3, ARA extract significantly increased the expression levels of PPARγ mRNA and protein. This data suggests that ARA extract promotes adipogenic differentiation via upregulation of PPAR γ expression.

### Effect of ARA extract on GLUT4 expression

**Figure 4:**
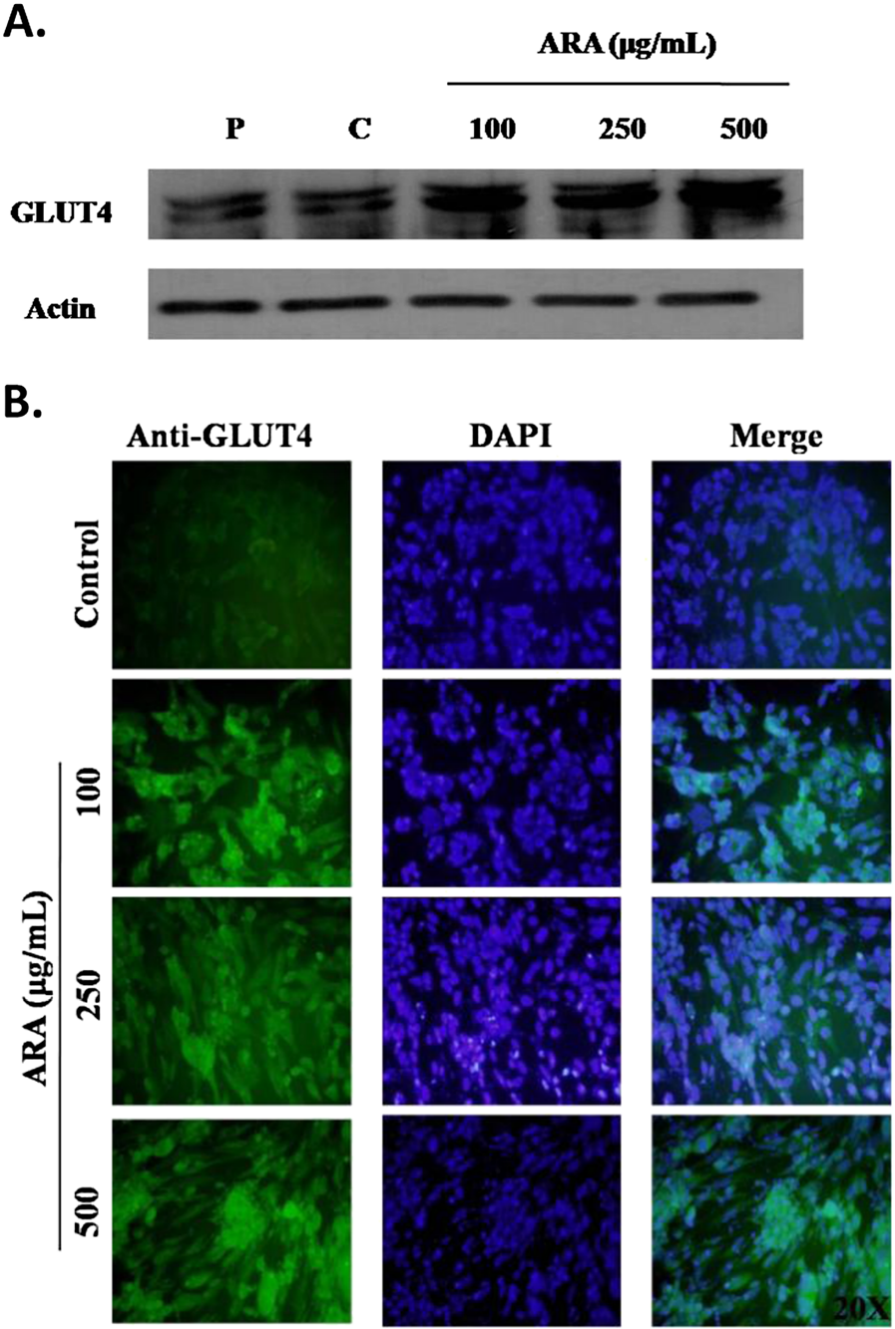
**Effect of ARA extract on GLUT4 expression in 3T3-L1 cells.** The cells were induced to differentiate into adipocytes with MDI medium with or without ARA (100, 200 and 500 mg/mL) extract for 8 days. (**A**) The expression of GLUT4 protein was analyzed by western blot. (**B**) Representative image of GLUT4-positive cells by immunostaining. Left panels, GLUT4-positive staining; middle panels, nuclear DAPI staining; and right panels, merged images of GLUT4 and DAPI. P: preadipocyte, C: control, 100: ARA 100 μg/mL, 250: ARA 250 μg/mL and 500: ARA 500 μg/mL.

GLUT4, an insulin-regulated glucose transporter, is highly expressed in adipose tissues [5]. Therefore, we investigated the expression of GLUT4 in 3T3-L1 cells by western blot and immunofluorescent staining. As shown in Figure 4A, ARA extract significantly increased the expression of GLUT4 in 3T3-L1 cells. In keeping with the increased protein expression of GLUT4, immunofluorescence data also showed a considerable increase in GLUT4 expression by ARA extract treatment in 3T3-L1 cells (Figure 4B).

### Effect of ARA extract on PI3K pathway

**Figure 5:**
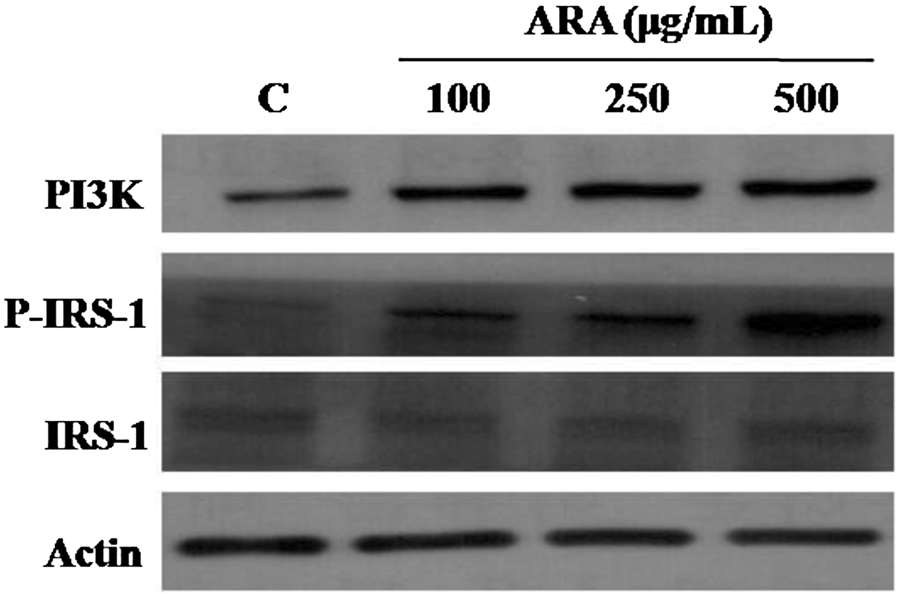
**Effect of ARA extract on PI3K/IRS-1 signaling pathway in 3T3-L1 cells.** The cells were induced to differentiate into adipocytes with MDI medium with or without ARA (100, 200 and 500 mg/mL) for 8 days. The expression of PI3K and the phosphorylation of IRS-1 were analyzed by western blot. Relative density was calculated as the ratio of PI3K and IRS-1 expression to actin expression. Each value represents the mean ± S.D. (*n* = 3). *p < 0.05 vs. control. C: control, 100: ARA 100 μg/mL, 250: ARA 250 μg/mL and 500: ARA 500 μg/mL.

The study evaluated the effects of ARA extract on the insulin signaling pathway in 3T3-L1 cells. Insulin signaling is initiated by the binding of insulin to the insulin receptor to activate IRS-1, which subsequently activates PI3K; the activation of PI3K results in the recruitment of GLUT4 to the cell surface [17]. In this study, the PI3K activation and IRS-1 phosphorylation were increased in ARA extract-treated cells in a concentration-dependent manner (Figure 5). This data demonstrated that ARA extract may improve glucose uptake in adipocytes by inducing GLUT4 expression and activation of the PI3K/IRS-1 signaling pathway.

## Discussion

The present study provides the first evidence that the root extract of *A. japonica* (ARA) promotes the insulin-induced conversion of preadipocytes into adipocytes, accompanied by increased expression of PPARγ, a key adipogenic transcription factor, in 3T3-L1 cells. Moreover, we have demonstrated that ARA extract activated the insulin signaling pathway by increasing GLUT4 and PI3K/IRS-1 phosphorylation in adipocytes. Our results suggest that ARA extract may have inhibitory activity against T2D by promoting adipogenic differentiation with activation of the insulin signaling pathway.

As an index of adipogenic differentiation, lipid accumulation was measured using Oil Red O staining because lipid droplets produced in the adipocyte cytoplasm are selectively stained with Oil Red O [18]. In this study, the effect of ARA extract on adipogenic differentiation was evaluated using Oil Red O staining. The result showed that treatment of ARA extract in 3T3-L1 preadipocytes remarkably promoted adipogenic differentiation. Furthermore, the enhanced rate of differentiation with ARA extract at 500 μg/mL was similar to that with troglitazone, a PPARγ activator. Troglitazone is a member of the drug class of TZDs (Thiazolidineodiones), which are a class of synthetic PPARγ ligands that improve insulin resistance in target tissues and are used for the treatment of T2D [13]. PPARγ has emerged as a key regulator of adipogenic differentiation in adipose tissue [19]. In mature adipocytes, PPARγ regulates insulin signaling, glucose and lipid metabolism [20]. Therefore, PPARγ is a major target for the treatment of various diseases, including diabetes, and has been an attractive target for new drug discovery [13,20]. This study showed that ARA extract effectively promoted adipogenic differentiation in preadipocytes by enhancing the PPARγ transcription levels. Based on the results of the present study, we suggest that the stimulatory effect of ARA extract on adipogenic differentiation may be mediated by up-regulation of PPARγ expression and activity.

PPARγ ligands are known to increase glucose transport by insulin-sensitive tissues by regulating the expression of several genes involved in glucose metabolism [11]. Insulin responsiveness is acquired during the maturation and differentiation of adipocytes and involves the expression of proteins responsible for the phenotypic functions of adipocytes, such as GLUT4, PI3K and IRS-1 [21]. On the other hand, in insulin-resistant states, signal transduction via the insulin receptor is impaired and activation of downstream targets such as IRS-1 and GLUT4 is decreased [22]. In this study, we demonstrated that ARA extract can increase the phosphorylation of IRS-1 and PI3K and the expression of GLUT4 in adipocytes, suggesting that ARA extract may improve insulin resistance.

## Conclusions

In conclusion, our results showed that ARA extract significantly promoted adipogenic differentiation in 3T3-L1 preadipocytes with up-regulation of PPARγ expression via activation of the GLUT4/PI3K/IRS-1 insulin signaling pathways. Although the possibility that other mechanisms of ARA extract improve diabetic conditions should be studied, our findings suggest that ARA extract is an attractive therapeutic agent for managing T2D.

## Competing interests

The authors declare that they have no competing interests.

## Author’s contributions

YKH designed research, HWJ and Y-KP conducted research. YKH and HWJ analyzed data. YKH wrote the manuscript. Y-KP had the primary responsibility for final content. All authors read and approved the final manuscript.

## Pre-publication history

The pre-publication history for this paper can be accessed here:

http://www.biomedcentral.com/1472-6882/12/154/prepub

## References

[B1] Prentki M Nolan CJ Islet β cell failure in type 2 diabetes J Clin Invest 2006 116 1802 1812 10.1172/JCI29103 16823478 PMC1483155

[B2] Zhou J Zhou S Berberine regulates peroxisome proliferator-activated receptors and positive transcription elongation factor b expression in diabetic adipocytes Eur J Pharmacol 2010 649 390 397 10.1016/j.ejphar.2010.09.030 20868663

[B3] Castan-Laurell I Dray C Knauf C Kunduzova O Valet P Apelin, a promising target for type 2 diabetes treatment? Trends Endocrinol Metab 2012 23 234 241 10.1016/j.tem.2012.02.005 22445464

[B4] Burén J Liu HX Lauritz J Eriksson JW High glucose and insulin in combination cause insulin receptor substrate-1 and −2 depletion and protein kinase B desensitization in primary cultured rat adipocytes: possible implication for insulin resistance in type 2 diabetes Eur J Endocrinol 2003 148 157 167 10.1530/eje.0.1480157 12534369

[B5] Xue P Hou Y Zhang Q Woods CG Yarborough K Liu H Sun G Anderson ME Pi J Prolonged inorganic arsenite exposure suppresses insulin-stimulated AKT S473 phosphorylation and glucose uptake in 3T3-L1 adipocytes: involvement of the adaptive antioxidant response Biochem Biophys Res Commun 2011 407 360 365 10.1016/j.bbrc.2011.03.024 21396911 PMC3086019

[B6] Ahn J Lee H Kim S Ha T Curcumin-induced suppression of adipogenic differentiation is accompanied by activation of Wnt/β-catenin signaling Am J Physiol Cell Physiol 2010 298 1510 1516 10.1152/ajpcell.00369.2009 20357182

[B7] Liu J Lin H Cheng P Hu X Lu H Effects of ghrelin on the proliferation and differentiation of 3T3-L1 preadipocytes J Huazhong Univ Sci Technol 2009 29 227 230 10.1007/s11596-009-0218-x 19399410

[B8] Kim CK Kim M Oh SD Lee SM Sun B Choi GS Kim SK Bae H Kang C Min BI Effects of Atractylodes macrophala Koidzumi rhizome on 3T3-L1 adipogenesis and an animal model of obesity J Ethnopharmacol 2011 137 396 402 10.1016/j.jep.2011.05.036 21669278

[B9] Zhang WY Lee JJ Kim IS Kim Y Park JS Myung CS 7-O-Methylaromadendrin stimulates glucose uptake and improves insulin resistance in vitro Biol Pharm Bull 2010 33 1494 1499 10.1248/bpb.33.1494 20823563

[B10] Xu C Wang LL Liu HY Zhou XB Cao YL Li S C33H, a novel PPARα/γ dual agonist, has beneficial effects on insulin resistance and lipid metabolism Acta Pharmacol Sin 2006 27 223 228 10.1111/j.1745-7254.2006.00263.x 16412273

[B11] Choi K Kim YB Molecular mechanism of insulin resistance in obesity and type 2 diabetes Korean J Intern Med 2010 25 119 129 10.3904/kjim.2010.25.2.119 20526383 PMC2880683

[B12] Lee SH Lee HJ Lee YH Lee BW Cha BS Kang ES Ahn CW Park JS Kim HJ Lee EY Korean red ginseng (panax ginseng) improves insulin sensitivity in high fat fed Sprague–Dawley rats Phytother Res 2012 26 142 147 10.1002/ptr.3610 22034219

[B13] Kim D Park KK Lee SK Lee SE Hwang JK Cornus kousa f. buerger ex miquel increases glucose uptake through activation of peroxisome proliferator-activated receptor gamma and insulin sensitization J Ethnopharmacol 2011 133 803 809 10.1016/j.jep.2010.11.007 21070843

[B14] Hong MH Kim JH Bae H Lee NY Shin YC Kim SH Ko SG Atractylodes japonica koidzumi inhibits the production of proinflammatory cytokines through inhibition of the NF-kappaB/IkappaB signal pathway in HMC-1 human mast cells Arch Pharm Res 2010 33 843 851 10.1007/s12272-010-0606-6 20607488

[B15] Wang KT Chen LG Wu CH Chang CC Wang CC Gastroprotective activity of atractylenolide III from Atractylodes ovate on ethanol-induced gastric ulcer in vitro and in vivo J Pharm Pharmacol 2010 62 381 388 20487223 10.1211/jpp.62.03.0014

[B16] Wang KT Chen LG Chou DS Liang WL Wang CC Anti-oxidative abilities of essential oils from Atractylodes ovate rhizome Evid Based Complement Alternat Med 2011 10.1093/ecam/neq006 PMC3135905 21799672

[B17] Lee OH Lee HH Kim JH Lee BY Effect of ginsenosides Rg3 and Re on glucose transport in mature 3T3-L1 adipocytes Phytother Res 2011 25 768 773 21520470 10.1002/ptr.3322

[B18] Ikarashi N Tajima M Suzuki K Toda T Ito K Ochiai W Sugiyama K Inhibition of preadipocyte differentiation and lipid accumulation by orengedokuto treatment of 3T3-L1 cultures Phytother Res 2012 26 91 100 10.1002/ptr.3493 21557367

[B19] Sharma AM Staels B Peroxisome proliferator-activated receptor gamma and adipose tissue-understanding obesity-related changes in regulation of lipid and glucose metabolism J Clin Endocrinol Metab 2007 92 386 395 17148564 10.1210/jc.2006-1268

[B20] Yang Y Shang W Zhou L Jiang B Jin H Chen M Emodin with PPARγ ligand-binding activity promotes adipocyte differentiation and increases glucose uptake in 3T3-L1 cells Biochem Biophys Res Commun 2007 353 225 230 10.1016/j.bbrc.2006.11.134 17174269

[B21] Cho KW Lee OH Banz WJ Moustaid-Moussa N Shay NF Kim YC Daidzein and the daidzein metabolite, equol, enhance adipocyte differentiation and PPARgamma transcriptional activity J Nutr Biochem 2010 21 841 847 10.1016/j.jnutbio.2009.06.012 19775880

[B22] Oliver E McGillicuddy F Phillips C Toomey S Roche HM The role of inflammation and macrophage accumulation in the development of obesity-induced type 2 diabetes mellitus and the possible therapeutic effects of long-chain n-3 PUFA Proc Nutr Soc 2010 69 232 243 10.1017/S0029665110000042 20158940

